# Algerian Prickly Pear Seed By-Products: Fatty Acids Composition, Antioxidant, Enzyme Inhibitory Activities towards Tyrosinase, Urease, α-Amylase, and Cholinesterase, along with the Ability to Protect from Thermal Protein Denaturation

**DOI:** 10.3390/ph17091145

**Published:** 2024-08-30

**Authors:** Nassiba Chafaa, Camelia Mosbah, Latifa Khattabi, Karima Malaoui, Wafa Zahnit, Mohamed El Amine Smaali, Faiza Houri, Yazid Medfouni, Khalid Mashay Al-Anazi, Ahmad Ali

**Affiliations:** 1 Laboratory of Natural Substances, Biomolecules and Biotechnological Applications, Department of Natural and Life Sciences, University of Larbi Ben M’hidi, Oum El Bouaghi 04000, Algeria; 2 Institute of Applied Science and Technology (ISTA), Ain M’lila, University of Larbi Ben M’hidi, Oum El Bouaghi 04000, Algeria; 3 Biotechnology Research Center (C.R.B.t), Constantine 25016, Algeria; 4 Laboratory of Valorization and Promotion of Saharan Resource (VPRS), Faculty of Mathematics and Matter Sciences, University of Kasdi Merbah, Ouargla 30000, Algeria; 5 Opuntia AURES SARL, Oum El Bouaghi 04000, Algeria; 6 Department of Zoology, College of Science, King Saud University, Riyadh 11451, Saudi Arabia; 7 Department of Life Sciences, University of Mumbai, Vidyanagari, Santacruz (East), Mumbai 400098, India; ahmadali@mu.ac.in

**Keywords:** *Opuntia ficus indica*, prickly pear, seed by-products, GC-MS analysis, antioxidant, thermal protein denaturation, tyrosinase, urease, α-amylase, cholinesterase

## Abstract

Prickly pear seed is a source of the most expensive oil in the world, which is rich in vitamins and polyunsaturated fatty acids. Its extraction generates a large quantity of press cake. These two by-products need to be valued. The current study aimed to assess the fatty acid composition of oil and the phytochemical composition of press cake. In addition, the antioxidant and the inhibition of thermal protein denaturation effects of both Algerian seed by-products were evaluated with their inhibitory action against the activities of urease, tyrosinase, α-amylase, and cholinesterase enzymes. The GC MS analysis result revealed the richness of our oil in linoleic (74%) and palmitic (13%) acids methyl esters, respectively. The chemical composition of press cake was characterized by a high value of dry matter (94.94 ± 0.05%), especially the carbohydrates (85.13 ± 0.94%). The results of antioxidant activity presented by IC_50_ and A_0.5_ ranged from 7.51 ± 0.03 to 88.10 ± 0.92 µg/mL. Furthermore, the IC_50_ values were 40.19 ± 1.21 and 61.18 ± 0.03 µg/mL in thermal protein denaturation assay, and ranging from 22.97 ± 0.72 to 385.99 ± 0.27 µg/mL for the inhibition of enzymatic activities. These results indicate that the studied oil can be one of the strongest oils for its impressive effects and also encourage us to reuse its press cake in feed livestock.

## 1. Introduction

*Opuntia ficus indica* (L.) *Mill*., commonly referred to as the nopal or prickly pear cactus, is a member of the dicotyledonous angiosperm Cactaceae family, which is composed of over 1500 distinct species of cacti. This family is considered to be one of the most charismatic plant families. Its geographical range includes Mexico, Latin America, South Africa, and Mediterranean countries. It is grown in arid areas as a significant source of nutrients and food [1,2,3,4]. Its numerous qualities, including those related to nutrition, medicine, and human health, increase its consumption. Furthermore, because of its potential and edible oil yield, which ranged from 5.8 to 13.6%, its use was recommended [5,6], and it is considered a new source of fruit oils [7].

Fruit seed oils are very interesting because they are highly unsaturated edible oils with the ability to scavenge free radicals and act as antioxidants [8,9]. Fruit seeds are widely discarded as waste. Actually, these seeds were used for the extraction of a good edible oil [9]. These edible oils are rich in polyunsaturated fatty acids, including linoleic acid and linolenic acid, which have many beneficial effects on human health [10]. The oils were extracted by both conventional and innovative methods. Conventional methods, including chemical and mechanical extraction, were largely used. Mechanical extraction (pressing or pressing cold) is the least expensive method because it requires no input of heat or organic solvents; only a mechanical power input is necessary. Pressing can be defined as a compression step to exude a liquid from a porous matrix [11].

Prickly pear seeds contain an appreciable amount of oil [12,13]. It contains a high level of polyunsaturated fatty acids [8,14,15]. This oil has been found to have many biological effects, such as antioxidant, antibacterial, antifungal, antiviral, and anticancer, as well as antidiabetic, anti-inflammatory, and anti-ulcer properties [16,17,18,19,20]. The waste from this extraction or press cake is rich in nutrients and phenolic compounds [16,17,18,19,20].

The extraction of prickly pear seed oil by chemical solvent was largely studied [7,12]; for this, the oil extracted by the press cold method with its press cake was the choice of our study.

For the first time, *Opuntia ficus indica* seed by-products were valorized with the aim of exploring their potential applications in the present study. The study evaluated the antioxidant and thermal protein denaturation protection activities of *Opuntia ficus indica* seed by-products to determine whether press cake could be utilized as livestock feed. Additionally, the goal of inhibiting tyrosinase activity was assessed to explore the potential use of the oil in cosmetic applications such as preventing skin hyperpigmentation. Furthermore, we investigated whether these by-products, particularly the press cake, could serve as therapeutic agents by evaluating their effects on enzymes such as urease, cholinesterase, and alpha amylase, which their hyperactivity is involved in ulcers, Alzheimer’s, and diabetes, respectively. Therefore, the key questions were as follows: do these by-products have the potential for use in the treatment of these diseases, and can they be utilized as cosmetic ingredients or livestock feed?

## 2. Results

### 2.1. Oil Fatty Acids Composition

#### 2.1.1. Oil Yield

The yield of extracted PPSO extracted using the press-cold method was 5.27 ± 0.25%.

#### 2.1.2. Fourier-Transform Infrared Spectroscopy (FT-IR)

**Figure 1:**
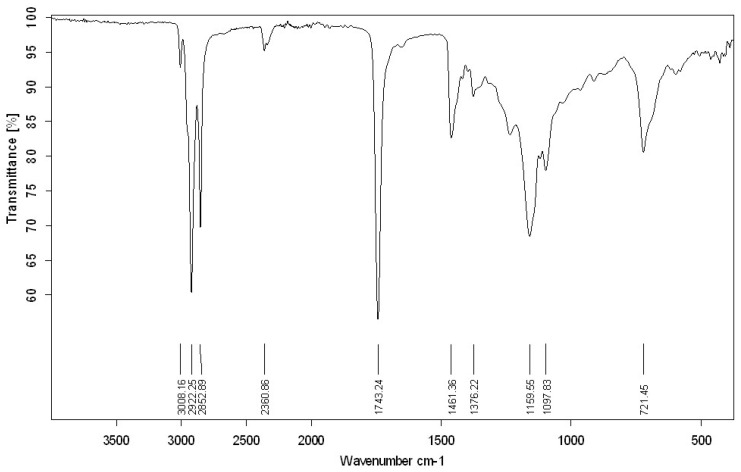
FT-IR analysis of prickly pear oil seeds.

As shown in Figure 1, the FT-IR spectra of PPSO indicated the presence of carbonyl with hydrogen bands, which confirms the presence of methyl esters in this sample.

#### 2.1.3. Gas Chromatography-Mass Spectrometry (GC-MS) Analysis

**Figure 2:**
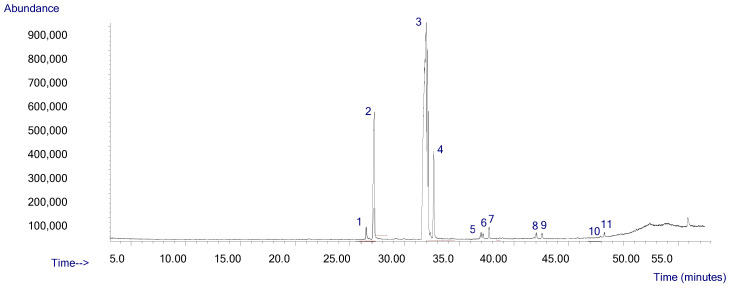
Chromatogram of GC-MS analysis of prickly pear seed oil.

In Figure 2, the GC-MS analysis of the PPSO sample revealed the presence of 11 peaks, which refers to 11 fatty acid methyl esters (FAMEs) identified in Table 1.

PPSO composition is characterized by a very high level of 9,12-octadecadienoic acid, methyl ester (linoleic acid, methyl ester) (74.24%), followed by hexadecanoic acid, methyl ester (palmitic acid, methyl ester) (13.08%), octadecanoic acid, and methyl ester (stearic acid, methyl ester) (6.75%), respectively. This PPSO including also many other FAMEs as traces: 9-hexadecenoic acid, 11-eicosenoic acid, 10-octadecenoic acid, heptadecanoic acid, 13-docosenoic acid, docosanoic acid, 15-tetracosenoic acid, and tetracosanoic acid methyl esters.

### 2.2. Phytochemical Composition of Press Cake

#### 2.2.1. Chemical Composition

The results (Table 2) of the chemical profile of PPPC powder demonstrated the presence of high dry matter contents, estimated at 94.94 ± 0.05%. This value indicates the low moisture level in our sample (5.45 ± 0.64%). The sample has a significant amount of ash content (1.54 ± 0.03%) and protein content (5.65 ± 0.04%). The examined PPPC also contains a notable quantity of fat (2.36 ± 0.10%). In addition, the sample has shown a high amount of carbohydrates (85.13 ± 0.94%) and an interesting value of energy equal to 384.46 ± 1.24 Kcal/100 g.

#### 2.2.2. Extraction of Phenolic Compounds

The yield of extraction using the method of maceration was important in hydroethanolic extract (4.29 ± 0.13%).

#### 2.2.3. Phenolic Content

The quantification of secondary metabolites demonstrated that the hydroethanolic extract contains important quantities of polyphenols, flavonoids, and flavonols. The results are shown in Table 3.

### 2.3. Biological Effects

#### 2.3.1. Antioxidant Activity

According to Table 4, PPSO has good IC_50_ values, which were 62.09 ± 0.68, 41.53 ± 0.17, and 88.10 ± 0.92 (µg/mL) for DPPH, ABTS, and β-carotene assays, respectively. On the other hand, the PPPC extract was more effective because it had lower IC_50_ values ranging from 21.74 ± 0.21 to 47.77 ± 0.51 µg/mL in the same assays. These values are higher than those of references BHT, BHA, and ascorbic acid. On the other hand, the A_0.5_ values of PPSO were 7.51 ± 0.03 and 52.13 ± 0.36 (µg/mL) against 23.51 ± 0.09 and 25.17 ± 0.19 µg/mL in phenanthroline and reducing power assays.

#### 2.3.2. Thermal Protein Denaturation Inhibition Assay

In this study, PPSO has an in vitro anti-inflammatory effect presented by an IC 50 value of 61.18 ± 0.03 µg/mL against 40.19 ± 1.21 and 43.12 ± 0.21 µg/mL of PPPC extract and diclofenac, respectively. The result is presented in Table 5.

#### 2.3.3. Inhibition of Enzymatic Activity

The results of the inhibition of enzymatic activities by our seed by-products are presented in Table 6.

#### Inhibition of Tyrosinase Activity

In this study, the inhibition of tyrosinase activity by our by-products increased with increasing concentrations, and the IC_50_ of PPSO was 40.49 ± 0.62 µg/mL against 138.29 ± 1.63 and 65.73 ± 0.14 µg/mL of PPPC extract and kojic acid, respectively. This result confirms that PPSO is a good protective product against melanogenesis disorder and can be used in cosmetics as a whitening product.

#### Inhibition of Urease Activity

The inhibition of urease activity was assessed by the indophenol method. This activity was measured by the inhibitory percentage and the conclusion of the IC_50_ values of PPSO, PPPC extract, and thiourea (as a reference). The IC_50_ values of both PPSO (10.91 ± 0. 01 µg/mL) and thiourea (8.42 ± 0.60 µg/mL) were very low against (132.62 ± 0.75 µg/mL) of the PPPC extract.

#### Inhibition of Alpha Amylase Activity

The results of this activity are presented in Table 3. The PPPC extract offered an inhibition of α-amylase activity with a lower IC_50_ value (368.86 ± 0.70 µg/mL) against (385.99 ± 0.27 μg/mL) for PPSO and (3431.01 ± 2.72 μg/mL) for acarbose, respectively. Our samples are more effective than the reference.

#### Inhibition of Cholinesterase Activity

In this activity, the PPPC extract has the higher IC_50_ values, which were 37.23 ± 1.76 to 368.86 ± 0.70 µg/mL in the inhibition of butyrylcholinesterase (BCHE) and acetylcholinesterase (ACHE) activities, respectively. The PPSO showed more important results presented by low IC_50_ values (22.97 ± 0.72 and 167.50 ± 0.62 µg/mL). Our samples are more active than the galantamine reference in inhibiting butyrylcholinesterase activity.

## 3. Discussion

The press-cold process is primarily used to extract prickly pear seed oil (PPSO), protecting the oil’s fatty acid and tocopherol concentrations while reducing oxidation. A previous study found that, the yield of Tunisian prickly pear oils extracted by cold press ranged from 7.1 to 11.4% [21]. In addition, for eight different prickly pear cultivars, the PPSO yield varied from 0.51 ± 0.01 to 6.1 ± 0.6 g/100 g when the same extraction method was used [22]. Other techniques were also used to extract this oil, such as maceration, Soxhlet, and maceration-percolation. Additional research investigated that the Soxhlet method produced the largest yield [6,23,24]. These findings confirmed that PPSO yield is based on plant origin and extraction method.

Fourier-transform infrared (FTIR) spectroscopy is an analytical method widely used in research laboratories and in the food industry that targets the characterization of vegetable and edible oils with specific bands or regions in the spectrum. It is a quick, nondestructive, and environmentally friendly technique [25,26].

For the GC-MS analysis, prior researchers found that the predominant fatty acid in PPSO from various cultivars was linoleic acid [7,12,27]. Our oil has the highest level of linoleic acid compared to previous investigations. Our result is in agreement with those of the precedent study [28], which confirmed that Moroccan *Opuntia ficus indica* and *Opuntia dillenii* oils (extracted by maceration) were mainly rich in linoleic and palmitic acids, respectively, with different concentrations. Another previous study investigated the FAME composition of *Opuntia dillenii* seed oil from an Iraqi cultivar [29]. This oil was extracted by a hydro-distillation process and included linoleic acid and palmitic acid methyl esters as major FAME at 72.90% and 15.12%, respectively. Recently, another study investigated the fatty acid composition of a Saudi Arabian PPSO [16], which was highly similar to our FAME composition. All these studies confirm that PPSO is richer in fatty acids and demonstrate that oil composition varies from a cultivar to another, and it is dependent on seed maturity, in addition to plant cultivar and oil extraction method.

PPSO contains many other classes of volatile compounds, such as aldehydes, alcohols, hydrocarbons, and ketones [30]. PPSO also contains other compounds, such as phenolic compounds, especially flavonoids, which are present in many prickly pear oils [31].

The chemical characterization of the prickly pear press cake (PPPC) sample, as shown in Table 1, demonstrates the presence of low moisture, which is particularly helpful for prolonging the shelf life of the samples and is a good predictor of the powder’s resistance to microbial contamination over an extended storage period [32,33]. These findings are in agreement with a precedent study based on prickly pear seed flour [24]. The sample has a significant amount of ash content, which is similar to those studied previously [21,24]. Protein content in PPPC is within the typical range for prickly pear seeds [34]. The examined PPPC also contains a notable quantity of fat, which is near that of the precedent study [21].

The primary component of our sample was carbohydrates. The main component of prickly pear seeds from Turkey cultivars is also carbohydrates [35]. The presence of carbohydrates in the sample is crucial, which is essential for the nervous system and basic metabolism to receive enough energy [36]. Moreover, certain carbohydrates help organisms make cartilage, mucus, nucleic glycoproteins, acids, and immunoglobulins [37]. Carbohydrates play a role in the balance of protein and fat metabolism, and they are required by the liver for the breakdown of fat [38]. Comparing our results to those of other fruits, date seed cake had 4.92% fat and 6.35% protein [39], which were slightly higher than our result. Based on all these results, it is clear that the chemical composition is influenced by the plant’s cultivar and its origin.

For extraction yield, our result is consistent with those reported in the literature for seeds of *Opuntia* spp., which range from 0.51 to 15.54% [22]. The extraction yield is impacted by the solvent and the extraction technique.

Phenolic compounds are secondary metabolites that contain many groups, such as flavonoids (the major group), phenolic acids, stilbenes, and tannins. Flavonoids are mostly found in plants as glycosylated derivatives, and they are responsible for the brilliant shades of orange, red, and blue seen in leaves, fruits, and flowers [40,41]. According to Table 2, hydroethanolic extract has a higher phenolic content than aqueous extract. Precedent studies demonstrated that solvent mixtures are stronger than pure solvents for phenol extraction from plants [42,43]. These results are in agreement with ours. However, the phenolic and flavonoid contents of the PPPC extracts of four *Opuntia* spp. from Tunisia were examined [21]. These molecules have high importance and have recently caught the attention of consumers as functional foods [44].

Many phenolic compounds of prickly pear seeds were previously determined, including feruloyl derivation, coumaric acid, amentoflavone, lupin isoflavone, myricetin, chlorogenic acid, kaempferol-3-O-rutinoside, isorhamnetin, protocatechuic acid, gallic acid, synaptic acid, and naringenin [12,45,46]. These phenolic compounds are protectors of plants against ultra-violet irradiation, pathogens, or predators. These compounds are also very important for human health [47,48].

Oxidative stress has now become a serious problem that is attracting the researchers’ attention. It is caused by an imbalance between antioxidants and free radicals and leads to cell molecule damage, such as carbohydrates, fats, proteins, and nucleic acids [49]. The antioxidant activity of PPSO and PPPC extract was measured by five methods: DPPH, ABTS, reducing power, β-carotene, and phenanthroline assays. These activities increase proportionally with sample concentrations. Many precedent studies confirmed the antioxidant activity of PPSO and PPPC extracts from many cultivars by many methods [20,21,42]. Phenolic acids and flavonoids are the main groups of bioactive molecules and secondary metabolites in plants [50]. They have an antioxidant substance capable of scavenging free superoxide radicals, being anti-aging, and reducing the risk of cancer [51]. The structure and substituents of the heterocyclic and B rings of flavonoids affect their ability to scavenge free radicals [52]. The presence of a catechol group in ring B, which has superior electron-donating qualities and is a radical target, and the conjugation of a 2,3-double bond with the 4-oxo group, which causes electron delocalization are the primary factors influencing radical-scavenging capability [53].

Reactive oxygen species (ROS) are by-products of regular cellular metabolism, typically generated at modest levels. They play essential roles in normal physiological functions within cells. However, when their production exceeds manageable levels, they can induce harmful modifications in cellular components like lipids, proteins, and DNA. This imbalance between oxidants and antioxidants, favoring the former, is termed “oxidative stress”. Such stress is implicated in various health concerns, including cancer, neurological disorders, hypertension, ischemia, diabetes, acute respiratory distress syndrome, and more [54]. Thankfully, antioxidants serve as regulators in this mechanism, either naturally produced within the body or supplied externally through diet and herbal supplements. Higher levels of antioxidants are associated with enhanced resilience against various health issues, underscoring their pivotal role in sustaining overall well-being [55].

Protein denaturation is caused by many agents, such as heat, solutions of acids or alkalis, and some other agents. This denaturation affects the isoelectric point, the structure, the solubility of albumin, and globulin proteins [56]. Furthermore, it has been stated that protein denaturation is a pathological process that results in a loss of functioning [57]. Moreover, many documents explain that one of the main causes of inflammation is protein denaturation. Inflammatory illnesses may benefit from the use of medicines that can stop protein denaturation [58]. However, the prevention of thermal protein denaturation is among their secondary actions, as shown in the literature [59,60], and they have been used as reference compounds in the thermal protein denaturation assay by several researchers [61,62,63,64].

The test of protection of thermal protein (BSA) denaturation was used to avoid the ethical problems surrounding the use of animals, particularly during the initial phases of searching for plants that may contain primary anti-inflammatory compounds. Our results indicate that the OFI by-products (PPSO and PPPC) have a good anti-denaturation protein effect. This finding motivates us to test confirmatory methods to assess the anti-inflammatory activity both in vivo and in vitro.

Many studies confirmed the anti-inflammatory effect of PPSO in vitro and in vivo [65,66,67], but no study was found highlighting the protection from thermal protein denaturation of PPPSO and PPPC by-products. Polyunsaturated fatty acids can enhance the synthesis of endothelial nitric oxide (NO) by decreasing the generation of TNF-α, IL-6, and the activation of NF-κB, which are essential steps in the inflammatory reaction [68,69]. Phenolic compounds also play a role in the regulation of inflammatory reactions [70].

Tyrosinase, a glycosylated and copper-containing oxidase enzyme, plays a crucial role in both fruit or fungal enzymatic browning and mammalian melanogenesis [71]. It is responsible for the oxidation of L-tyrosine or L-3,4-dihydroxyphenylalanine (L-DOPA) to DOPA-quinone, which is the rate-limiting step in melanin biosynthesis [72]. Tyrosinase’s inhibitory activity can be determined by measuring the amount of dopachrome produced when the enzyme substrate, L-DOPA, is present [73].

Tyrosinase activity is based on the composition of PPSO, which is rich in polyunsaturated fatty acids. A previous study explained that unsaturated fatty acids such as linoleic and palmitic acids are intrinsic factors that regulate the degradation of membranous glycoproteins such as tyrosinase [74]. On the other hand, certain phenolic compounds are presented as tyrosinase inhibitors [75,76]. PPSO and PPPC can be used for whitening skin.

The inhibition of tyrosinase activity of Algerian and universal oils is widely assessed [77,78,79,80,81]. On the other hand, the inhibition of this activity by PPSO and PPPC is assessed for the first time in this study.

Diverse bacteria, fungi, algae, and plants have a urease enzyme, also known as urea amidohydrolase.

In the final stage of nitrogen metabolism in living things, this enzyme catalyzes the hydrolysis of urea into ammonia and carbamate [82]. This enzyme plays an essential role in the appearance of ulcers and cancers. It facilitates the colonization of *Helicobacter pylori* in the acidic environment of the stomach, which leads to the appearance of many chronic diseases [83]. Many previous studies confirmed the ability of oils and extracts of plants to inhibit the urease activity of many oils [84,85,86]. Based on precedent studies, fatty acids, terpenoids, and alkanes inhibit the urease enzyme and the activity of *H. pylori* [87,88,89,90]. Inhibiting urease activity is thought to be a viable ulcer therapy since it can stop the *H. pylori* infection, which touches 50% of the population [91,92]. To the best of our knowledge, there have been no reports in the literature on the inhibition of urease activity by *Opuntia ficus indica* seed oil and press cake.

The pancreatic α-amylase enzyme is responsible for the digestion of starch to α-dextrin or oligosaccharides, which were transformed by intestinal α-glucosidase to simple sugar “glucose” before its absorption by the duodenum and upper jejunum [93,94]. The inhibition of the α-amylase enzyme and α-glucosidase by synthetic or natural molecules is key to the control of diabetes type 2 by decreasing the postprandial glycemia level [95,96,97,98]. Many precedent studies confirmed that *Opuntia ficus indica* and *Opuntia dillenii* seed oils play a role in the regulation of blood glucose levels in vivo and have an antidiabetic effect in vivo [18,99]. The main components of plant seed oil are the fatty acids, which enter human physiology [100]. Polyunsaturated fatty acids (PUFAs) are known for their nutritional value [101]. In addition, many authors confirmed that FAs from seed oils are excellent inhibitors of the α-amylase enzyme. Moreover, both ω-6 and ω-3 play a role in the regulation of many physiologic mechanisms in the liver and in skeletal muscle, which is related to diabetes [102,103,104]. The phenolic compounds are known for their antioxidant potential, and many studies have confirmed that the phenolic compounds also play a role as digestion enzyme inhibitors. A previous study reported that the tannin content of fruit extracts has the potential to inhibit both salivary and pancreatic amylase enzymes [105]. These natural phenolic compounds can be candidates as safer alternatives to synthetic molecules of diabetes 2, such as acarbose, which is hepatotoxic.

Neurodegenerative diseases are expressed as the loss of neurons in the central nervous system, which leads to the dysfunction of this part of the brain. The main causes of these diseases are Alzheimer’s and Parkinson’s disease [70]. In our study, the inhibition of cholinesterase activity was based on the reaction of plant extracts with both acetylcholinesterase and butyrylcholinesterase. The results of this activity are very interesting, indicating that the prickly pear seed oil and the hydroethanolic extract of the press cake can act as inhibitors of the activity of cholenisterase enzymes. These results are represented for the first time. Many phenolic compounds were investigated as cholinesterase inhibitors, such as gallic acid, chlorogenic acid, and ferulic acid [106]. These molecules were previously found in prickly pear seed extracts [12,46]. The phenolic acids can be used as preventive nutrients for Alzheimer’s disease [107]. Other flavonoids, such as quercetin and myricetin, have an inhibitory effect against cholinesterase enzymes [108]. Many authors have investigated the anticholinesterase activity of many oils [86,109].

In general, the inhibition of enzymatic activities by PPSO is due to the presence of not only fatty acids but also flavonoids, which are responsible for healthcare effects. At the same time, the phenolic compounds of PPPC extract are also responsible for their biological effects.

The biological activities of other seed oils were compared with our PPSO in Table 7. Indeed, the antioxidant activity was better than that of *Crotalaria juncea Linn* and approximately that of *Nigella sativa.* The anti-inflammatory effect of PPSO is more powerful than the *Nigella sativa and Monodora myristica* effects but lower than the effect *of Argania spinose.*

In addition, the inhibition of enzymatic activities of PPSO was more potent than other oils (Table 7), except for the inhibition of acetylcholinesterase, where *Nigella sativa* was more effective.

## 4. Materiel and Methods

Prickly pear seeds were supplied by an agro-industrial processing unit (Opuntia Aures) located in Oum El Bouaghi in southeast Algeria in May 2023. The oil of these seeds was extracted by cold pressing, weighted, yielded, and conserved in small dark glass vials for further analysis. The recovered prickly pear press cake (PPPC) was dried, ground into a fine powder using a coffee grinder, and stored for further analysis.

### 4.1. Oil Fatty Acids Characterization

The fatty acid composition was revealed after three essential steps: the preparation of fatty acid methyl ester (FAME), FT-IR analysis, and chromatographic analysis.

#### 4.1.1. Preparation of Fatty Acids Methyl Ester (FAMEs)

FAMEs were obtained from the triglycerides of oil by the transesterification reaction, treating 0.10 g of oil with 0.2 mL of a 2 N of KOH solution in MeOH. The resulting FAME solution was extracted with 2 mL of *n*-hexane. This fraction was then diluted 1:20 with *n*-hexane before its injection into the gas chromatography-mass spectrometry (GC-MS) apparatus.

#### 4.1.2. Fourier Transform Infrared Spectroscopy (FTIR)

FTIR spectra of the fatty acid methyl ester were recorded using an FTIR spectrometer alpha II (BRUKER, Berlin, Germany) in the range of 3500–500 cm^−1^ with a resolution of 4 cm^−1^.

#### 4.1.3. Gas Chromatography Mass Spectrometry (GC-MS) Analysis

The fatty acid composition of the extracted oil was determined by gas chromatography-mass spectrometry (GC-MS). The analysis of methylated fatty acids was performed on a Hewlett-Packard Agilent 6890 plus quadrupole gas chromatography-mass spectrometer (GC-MS) instrument equipped with a carbowax (30 m × 0.25 mm ID; 0.25 μm film thickness) capillary. In total, 0.2 µL of the sample was injected into the capillary column. Helium was used as the carrier gas. Injector and detector temperatures were set at 250 °C. The injection was performed in split mode (1:50). The column temperature was programmed initially at 70 °C for 5 min, then to increase at a rate of 5 °C per minute at the final temperature of 280 °C. Fatty acid methyl esters were separated at a constant pressure (100 kPa), and peaks were identified by comparing the mass spectra with the mass spectral database. The identification of compounds was based on the comparisons of their mass spectra with the NIST Library 2008. Data were expressed as individual fatty acid methyl esters percentages in the lipid fraction.

### 4.2. Phytochemical Composition of Press Cake

#### 4.2.1. Chemical Composition

The ground sample of PPPC was examined for moisture, ash, crude fat, and crude protein contents [118].

Total carbohydrates (% TCH) were calculated by the following formula:

% TCH = 100 − SA(1)

SA = % H_2_O + % protein + % fat + % ash (2)

Energy Value

The energy value of the PPS and PPPC samples was estimated by multiplying the percentages of crude protein, crude fat, and carbohydrates by factors of 4, 9, and 4, respectively [119].

Energy (kcal/100 g) = (% protein × 4) + (% fat × 9) + (% carbohydrates × 4).

#### 4.2.2. Extraction Procedure

Fifty grams of PPPC were macerated for 24 h with constant stirring in a hydroalcoholic solution of ethanol (80%) and then filtered through Wattman paper. The marc was used for another extraction with a new quantity of ethanol 80% for 24 h and filtrate. The two filtrates were mixed and dried at 38 °C in a rotary evaporator. After being yielded, the extracts were kept for later examination at 4 °C in sterile containers.

#### 4.2.3. Phytochemical Composition

##### Total Phenolic Content

Total phenolic content is determined by the Folin-Ciocalteu [120]. To 20 µL of PPPC extract or methanol (for blank), 100 µL of diluted FCR (1:10) was added, followed by 75 µL of sodium carbonate (7.5%). The reaction was left to incubate in the dark for 2 h. The reading was taken at 765 nm using a multi-microplate reader (Perkin Elmer, Enspire, Waltham, MA, USA). Gallic acid was used as a standard, and a calibration curve was plotted in a concentration range of 0 to 200 µg/mL. All analyses were performed in triplicate, and phenolic compound content was expressed as µg of gallic acid equivalent (GAE)/mg.

#### Flavonoid Content

The determination of the flavonoid content of extracts was based on the formation of a complex between aluminum trichloride and flavonoids. The mixture reaction contains 100 µL of PPPC extract and 100 µL of 2% aluminum chloride (AlCl_3_ in ethanol). The mixture was incubated for 15 min and read at 415 nm using a microplate reader. Quercetin was used as a standard, and a calibration curve was plotted in a concentration range of 0 to 250 µg/mL [121]. All analyses were performed in triplicate, and flavonoid content was expressed as µg of quercetin equivalent (QE)/mg of extract.

#### Flavonol Content

The determination of flavonol content was determined according to the Kumaran procedure [122]. The mixture reaction contains 50 µL of PPPC extract, 50 µL of 0.2% aluminum chloride (AlCl_3_ in ethanol), and 50 µL of sodium acetate. The mixture was incubated for 180 min, and the absorbance was measured at 440 nm using a microplate reader. Quercetin was used as a standard, and a calibration curve was plotted in a concentration range of 0 to 250 µg/mL. All analyses were performed in triplicate, and flavonoid content was expressed as µg of quercetin equivalent (QE)/mg of extract.

### 4.3. Biological Activities

#### 4.3.1. Antioxidant Activity

##### 2,2-Diphényl 1-Picrylhydrazyle (DPPH) Assay

DPPH activity was measured using the protocol of Blois [123], with minor modifications. In brief, we added 40 µL of diluted PPSO, PPPC extract, or references (BHT, BHA, and ascorbic acid) at different concentrations to 160 µL of DPPH solution (4% in methanol) in a 96-microplate well. After incubation for 20 min, the absorbance was measured at 517 nm using a microplate reader. This activity was repeated three times. The result is expressed as the IC_50_ value, which is the sample concentration required to inhibit 50% of DPPH molecules.

#### 2,2′ Azion-bis 3-Ethylbenzothizoline-6-sulfonic Acid (ABTS) Assay

The ABTS (2,2′ azion-bis 3-ethylbenzothizoline-6-sulfonic acid) assay was performed using the standard methodology [124] based on the discoloration kinetics of the ABTS ion. ABTS powder was dissolved at a concentration of 7 mM in 5 mL of distilled water and mixed with an equal volume of potassium persulfate solution (2.45 mM in water). The mixture was incubated for 12 to 16 h at room temperature in the dark. Before use, the ABTS solution was diluted with water until the absorbance at 734 nm was 0.700 ± 0.02. A microplate well was prepared by mixing 40 μL of diluted PPSO, PPPC extract, or references (BHT, BHA, and ascorbic acid) at different concentrations with 160 µL of ABTS solution and incubating for 10 min at room temperature in the dark. The absorbances were measured at 734 nm. This activity was repeated three times. The result is expressed as IC_50_ values, which are the sample concentrations required to inhibit 50% of ABTS molecules.

#### Reducing Power Assay

This assay was assessed by the standard method with minor modifications [125]. In a microplate well, we put 10 µL of diluted PPSO, PPPC extract, or references (BHT, BHA, and ascorbic acid) at different concentrations with 40 µL of phosphate buffer (0.2 M; pH = 6.6), followed by 50 µL of 1% potassium hexacyanoferrate [K_3_Fe_2_(CN)_6_]. This plate was immediately incubated at 50 °C for 20 min. After incubation, 50 µL of 10% trichloroacetic acid was added, followed by 40 µL of distilled water, and finally, 10 µL of a 0.1% FeCl_3_ solution. The absorbance was directly measured at 700 nm. Reducing power was expressed as A_0.5_, which is the sample concentration required to give an absorbance of 0.5.

#### Phenanthroline Assay

This assay was assessed according to the precedent method of Szydłowska-Czerniak et al. [126]. Briefly, a mixture of 10 µL of diluted PPSO, PPPC extract, or references (BHT, BHA, and ascorbic acid) at different concentrations, 50 µL of FeCl_3_ (0.2%), 30 µL of phenanthroline (0.5%), and 110 µL of MeOH was prepared in a microplate well and incubated immediately in an oven at 30 °C for 20 min. Then, the absorbance of precedent reactions was measured at 510 nm by a microplate reader. This assay was expressed as A_0.5_, which is the sample concentration required to give an absorbance of 0.5.

#### β-Carotene Assay

The β-carotene assay was measured according to the protocol of Marco [127], with minor modifications. For the preparation of β-carotene solution, 0,5 mg of β-carotene was added to the mixture of 200 µL Tween 40, 25 µL linoleic acid, and 1 mL of chloroform solvent. This solvent was evaporated at 40 °C under vacuum. Finally, fifty mL of oxygenated ultra-pure water was added (a water bubble with oxygen for 30 min at a flow rate of 100 mL/min), and this solution was vigorously shaken. Next, the absorbance of this solution was measured and adjusted to 0.9. In a microplate well, 40 µL of diluted PPSO, PPPC extract, or references (BHT, BHA, and ascorbic acid) at different concentrations was added to 160 µL of B-carotene solution, and the absorbance was measured at 0 and 120 min at 470 nm, and the incubation was at 45 °C. The IC_50_ values of PPSO, PPPC extract, and standards (BHT, BHA, and ascorbic acid) were measured.

#### 4.3.2. Thermal Protein Denaturation Inhibition Assay

This activity is based on the ability of our by-products to inhibit the denaturation of BSA (bovine serum albumin) caused by heating (at 72 °C) [62]. 100 µL of diluted PPSO, PPPC extract, or references (ibuprofen and diclofenac) at different concentrations (0–2000 µg) were combined with 100 µL of BSA (0.2%, in Tris-HCl, pH: 6.6). The mixtures were kept at 37 °C for 15 min and heated to 72 °C in a water bath for five minutes. After cooling, the turbidity was measured using a microplate reader at 660 nm. The blank of each concentration contained 100 µL of samples with 100 µL of BSA. The experiment with each concentration was carried out in triplicate, and the results are presented as IC_50_ values.

#### 4.3.3. Inhibition of Enzymes Activities

##### Inhibition of Tyrosinase Activity

The inhibition of this activity was measured by the spectrophotometric method [128]. The enzyme was extracted from mushrooms, and L-DOPA was used as a reaction substrate (1 mg/mL). Briefly, 20 µL of enzyme solution was added to 150 µL of sodium phosphate buffer (0.1 M, PH 6.8) and 10 µL of PPSO, PPPC extract, or standard (Kojic acid), then incubated for 10 min at 37 °C. In addition, 20 µL of L-DOPA was added to the reaction, and the microplate was incubated again for 10 min at 37 °C. The absorbance was reading at 475 nm.

#### Inhibition of Urease Activity

This activity was assessed by the standard approach method using the indophenol method [129]. In a 96-well microplate, 10 µL of the PPSO, the PPPC extract, or the reference (thiourea), diluted in methanol at various concentrations, were combined with 25 µL of urease solution (5 U/milliliter of urease from Jack bean *Canavalia ensiformis*, type IX), followed by the addition of 50 microliters of urea solution (17 mM), 45 μL of phenol reagent (8% phenol and 0.1% *w*/*v* sodium nitroprusside), and 70 μL of alkaline reagent (2.85% NaOH and 4.7% active chloride NaOCl), respectively. The microplate was incubated for 50 min at 30 °C, and the absorbance was measured at 630 nm. The result of this activity is reported as the IC_50_ value.

#### Inhibition of Alpha Amylase Activity

The α-amylase inhibition activity was measured using the starch-iodine test according to the method of Zengin et al. [130], with slight modifications. In a microplate well, 50 µL of α-amylase enzyme (1 mg/mL) was diluted in sodium phosphate buffer (0.1 M) and was added to 25 µL of PPSO, PPPC extract, or acarbose (as a reference) at different concentrations, and the mixture was incubated at 37 °C for 10 min. Next, 50 μL of 0.1% w/v soluble starch was added to the sample wells, and the microplate was incubated again for 10 min at 37 °C. After incubation, 25 μL of 1 M HCl was added to the reaction to halt the enzymatic process, followed by the addition of 100 μL of iodine reagent (IKI) (5 mM I2 and 5 mM KI). The absorbance was measured at 620 nm.

Inhibitory percentage (%) = 1 − [(A_c_ − A_e_) − (A_s_ − A_b_)/(A_c_ − A_e_)]

A_c_ = Absorbance of [35 µL of methanol + 50 µL of sodium phosphate buffer + 50 µL of starch solution + 25 µL of HCl + 100 µL of IKI]

A_e_ = Absorbance of [35 µL of methanol + 50 µL of enzyme + 50 µL of starch + 25 µL of HCL + 100 µL of IKI]

A_s_ = Absorbance of sample = [35 µL of sample + 50 µL of enzyme + 50 µL of starch + 25 µL of HCL+ 100 µL of IKI]

A_b_ = Absorbance of Blank of sample = Absorbance [35 µL of sample + 125 µL of buffer + 100 µL of IKI].

#### Inhibition of Cholinesterase Activity

Using the Ellman method [131], the original approach method was used to evaluate this activity. In microplate wells, 10 µL of PPSO, PPPC extract, or galantamine (as a reference), diluted in ethanol at different concentrations, were mixed with 150 µL of 100 mM sodium phosphate buffer (pH 8.0) and 20 µL of acetylcholinesterase (5.32 × 10^−3^ U) or butyrylcholinesterase (6.85 × 10^−3^ U) solutions and incubated for 15 min. Next, we added 10 µL of DTNB (0.5 mM), followed by 10 µL of acetylthiocholine iodide (0.71 mM) or 10 µL of butyrylthiocholine chloride (0.2 mM). The absorbance at 412 nm was measured for T = 0 min and T = 15 min after the incubation of the microplate for 15 min at 37 °C. The IC_50_ values were determined for the PPPC extract and the galantamine reference.

### 4.4. Statistical Analysis

The outcomes are presented as the mean values with a standard deviation (mean ± SD) based on three measurements. The IC_50_ and A_0.5_ values were determined through linear regression analysis, while variance analyses were conducted via ANOVA using XL STAT, version 2016.02.28451. Distinctions between means were assessed using the Tukey test, with significance defined as a *p* value < 0.001.

## 5. Conclusions

*Opuntia ficus-indica* (L.) *Miller* is one of the charismatic plants known in traditional medicine for its beneficial characteristics and nutritional values. The prickly pear has been the subject of several studies. Recently, many studies have targeted their seed by-products. In this study, we aimed to valorize both *Opuntia ficus indica* (OFI) seed by-products—oil and press cake—which were found to possess a significant chemical composition rich in fatty acids, flavonoids, and phenolic compounds. These by-products demonstrated potent antioxidant properties, confirmed through five methods: DPPH, ABTS, reducing power, β-carotene, and phenanthroline assays. Furthermore, both by-products exhibited strong inhibition of thermal protein denaturation. The antioxidant activity and inhibition of thermal protein denaturation suggest that these by-products can be effectively used as food additives, particularly in livestock feed.

Moreover, this study was the first to assess the inhibition of enzymatic activities by these by-products, yielding excellent results—especially for seed oil in the butyrylcholinesterase assay. This opens up the potential for using OFI seed oil in cosmetic products and as a general healthcare agent, with applications in preventing physiological dysfunctions related to enzyme hyperactivity, such as diabetes, ulcers, and Alzheimer’s disease. As for the press cake, we propose that it could serve as a valuable source of natural antioxidants or as livestock feed, thereby contributing to waste reduction.

## Figures and Tables

**Table 1:** FAMEs of prickly pear seed oil revealed by GC-MS analysis.

Peak	Retention Time (min)	Molecules	Area %
1	26.796	9-Hexadecenoic acid, methyl ester	1.31
2	27.584	Hexadecanoic acid, methyl ester	13.08
3	32.791	9,12-Octadecadienoic acid, methyl ester	74.26
4	33.591	Octadecanoic acid, methyl ester	6.75
5	38.312	11-Eicosenoic acid, methyl ester	0.84
6	38.524	10-Octadecenoic acid, methyl ester	046
7	39.113	Heptadecanoic acid, methyl ester	0.91
8	43.851	13-Docosenoic acid, methyl ester	1.23
9	44.434	Docosanoic acid, methyl ester	0.54
10	49.515	15-Tetracosenoic acid, methyl ester	0.25
11	49.852	Tetracosanoic acid, methyl ester	0.36

**Table 2:** Chemical composition of prickly pear press cake.

Parameters (%)	PPPC
Dry matter	94.94 ± 0.05
Moisture	5.45 ± 0.64
Ash	1.54 ± 0.03
Protein	5.65 ± 0.04
Fat	2.36 ± 0.10
Carbohydrates	85.13 ± 0.94
Energy value (Kcal/100 g)	384.46 ± 1.24

All values are expressed as Mean ± standard deviation (*n*= 3).

**Table 3:** Quantification of phenolic, flavonoid, and flavanol contents of PPPC extract.

Total Phenolic (µg GAE/mg)	Total Flavonoids (µg QE/mg)	Total Flavonols (µg QE/mg)
187.94 ± 0.48	63.43 ± 0.49	26.73 ± 0.29

All values are expressed as Mean ± standard deviation (*n* = 3).

**Table 4:** The results of antioxidant activity of prickly pear seed by-products.

	DPPH	ABTS	β-Carotene	Phenanthroline	Reducing Power
	IC_50_ (µg/mL)			A_0.5_ (µg/mL)	
PPSO	62.09 ± 0.68 ^a^	41.53 ± 0.17 ^a^	88.10 ± 0.92 ^a^	7.51 ± 0.03 ^b^	52.13 ± 0.36 ^a^
PPPC extract	31.25 ± 0.13 ^b^	21.74 ± 0.21 ^b^	47.77 ± 0.51 ^b^	23.51 ± 0.09 ^a^	25.17 ± 0.19 ^b^
BHT *	18.05 ± 0.20 ^d^	2.01 ± 0.03 ^d^	38.00 ± 0.52 ^d^	5.88 ± 0.14 ^c^	23.82 ± 0.06 ^c^
BHA *	11.23 ± 0.28 ^e^	1.73 ± 0.23 ^e^	33.62 ± 0.12 ^e^	2.60 ± 0.03 ^e^	15.33 ± 0.55 ^d^
Ascorbic acid *	22.01 ± 0.48 ^c^	3.45 ± 0.10 ^c^	45.82 ± 0.67 ^c^	2.70 ± 0.17 ^d^	11.33 ± 0.06 ^e^

All values are expressed as Mean ± standard deviation (*n*= 3). *: Reference compounds. IC_50_ and A_0.5_ values are defined as the concentration of 50% inhibition percentages and the concentration at 0.50 absorbance, respectively. IC_50_ and A_0.5_ were calculated by linear regression analysis and expressed as the mean ± SD (*n* = 3). The values with different superscripts (a–e) in the same line are significantly different (*p* < 0.001).

**Table 5:** IC_50_ (µg/mL) values of thermal protein denaturation inhibition activity of prickly pear seed by-products.

PPSO	PPPC	Ibuprofen	Diclofenac
61.18 ± 0.03 ^a^	40.19 ± 1.21 ^c^	43.12 ± 0.21 ^b^	13.28 ± 0.13 ^d^

IC_50_ values are defined as the concentration of 50% inhibition percentages. IC_50_ was calculated by linear regression analysis and expressed as the mean ± SD (*n* = 3). The values with different superscripts (a–d) in the same Line are significantly different (*p* < 0.001).

**Table 6:** IC_50_ values (µg/mL) of the inhibition of enzymatic activities by prickly pear seed by-products.

Inhibition Activity	Tyrosinase	Urease	α-Amylase	ACHE	BCHE
PPSO	40.19 ± 1.21 ^b^	10.95 ± 0.08 ^b^	385.99 ± 0.27 ^b^	167.50 ± 0.62 ^b^	22.97 ± 0.72 ^c^
PPPC extract	138.29 ± 1.63 ^a^	132.62 ± 0.75 ^a^	368.86 ± 0.70 ^c^	191.82 ± 1.43 ^a^	37.23 ± 1.76 ^a^
* Kojic acid	13.28 ± 0.13 ^c^	NT	NT	NT	NT
* Thiourea	NT	8.42 ± 0.06 ^c^	NT	NT	NT
* Acarbose	NT	NT	3431.01 ± 2.72 ^a^	NT	NT
* Galantamine	NT	NT	NT	79.66 ± 0.55 ^c^	46.48 ± 0.72 ^b^

*: reference compounds. IC_50_ values are defined as the concentration of 50% inhibition percentages. IC_50_ was calculated by linear regression analysis and expressed as the mean ± SD (*n* = 3). The values with different superscripts (a, b, and c) in the same line are significantly different (*p* < 0.001). NT: non tested.

**Table 7:** Comparison of PPSO results with other oils.

	Antioxidant (DPPH)	Thermal Protein Denaturation Inhibition	Tyrosinase Inhibition	Urease Inhibition	α-Amylase Inhibition	ACHE Inhibition	BCHE Inhibition
Our PPSO	62.09 ± 0.68	61.18 ± 0.03	40.19 ± 1.21	10.95 ± 0.08	385.99 ± 0.27	167.50 ± 0.62	22.97 ± 0.72
*Crotalaria juncea Linn* [110]	122.52	^-^	^-^	^-^	^-^	-	-
*Nigella sativa* [111,112,113]	52.61 ± 0.22	113.37	544.6 ± 1.915	30.21 ± 037	-	7.32 ± 0.41	35.48 ± 0.83
*Sideritis albiflora* [114]	ND	-	ND	-	-	ND	157.2 ± 0.9
*Sideritis leptoclada* [114]	ND	-	ND	-	-	ND	199.0 ± 1.0
*Argania spinosa* [115,116]	-	1.23 ± 0.75	-	-	780 ± 0.16	-	-
*Monodora myristica* [117]	^-^	258	-	-	-	-	-

ND: not detected.

## Data Availability

The data provided in the current paper are available upon request from the corresponding author.
